# Hybrid Clustering-Enhanced Brain Storm Optimization Algorithm for Efficient Multi-Robot Path Planning

**DOI:** 10.3390/biomimetics10060347

**Published:** 2025-05-26

**Authors:** Guangping Qiu, Jizhong Deng, Jincan Li, Weixing Wang

**Affiliations:** School of Artificial Intelligence, Zhujiang College of South China Agricultural University, Guangzhou 510900, China; jz-deng@scau.edu.cn (J.D.); xingong@scauzj.edu.cn (J.L.); weixing@scau.edu.cn (W.W.)

**Keywords:** multi-robot path planning, Brain Storm Optimization (BSO), hybrid clustering, path conflict avoidance

## Abstract

To address the core challenges in multi-robot path planning (MRPP) within large-scale, complex environments—namely path conflicts, suboptimal task allocation, and computational inefficiency—this paper introduces a Hybrid Clustering-Enhanced Brain Storm Optimization (HC-BSO) algorithm designed to improve both path quality and computational efficiency significantly. For optimizing initial task assignment, the conventional K-Means clustering method is supplanted by a hybrid clustering methodology that integrates Mini-Batch K-Means with Density-Based Spatial Clustering of Applications with Noise (DBSCAN), facilitating an efficient and robust partitioning of task points. Concurrently, we incorporate a two-stage exploration–perturbation evolutionary strategy. This strategy effectively balances global exploration with local exploitation, thereby enhancing solution diversity and search depth. Comparative analyses against the standard Brain Storm Optimization (BSO) and other prominent swarm intelligence algorithms reveal that HC-BSO exhibits significant advantages in terms of total path length, computational time, and path conflict avoidance. Notably, in large-scale, multi-task scenarios, HC-BSO consistently generates high-quality, conflict-free paths, demonstrating superior stability, convergence, and scalability.

## 1. Introduction

Rapid advancement in robotics and artificial intelligence has placed powerful multi-robot systems (MRS) as the cornerstone technology in diverse fields such as smart manufacturing, intelligent warehousing, logistics, environmental monitoring, and disaster relief [1,2,3,4]. Leveraging the collaborative capabilities of multiple robots, MRS exhibits superior efficiency, robustness, and flexibility compared to single-robot systems. Within these contexts, MRPP is pivotal for orchestrating efficient collaboration; its primary objective is to generate conflict-free and efficient paths for multiple robots, thereby maximizing system performance and task completion rates [5,6].

Despite considerable advancements in MRPP research, its inherent NP-hard nature poses significant challenges to achieving optimal planning, particularly in complex scenarios. First, path conflicts, such as collisions and deadlocks, severely threaten system stability and task success rates, representing a critical hurdle that MRPP must address [7,8]. Second, as the number of robots and task density increase, the dimensionality of the solution space escalates dramatically. This leads to high computational complexity and diminished solution efficiency, making it challenging to meet the demands of real-time applications. Furthermore, the need to rapidly adjust paths while maintaining planning quality in dynamic and complex task environments places greater demands on algorithmic robustness and adaptability [9,10,11]. Collectively, these challenges necessitate MRPP algorithms that not only guarantee conflict-free and optimal paths (e.g., minimizing total path length) but also strike a balance between computational efficiency and robust responsiveness to environmental variations.

In recent years, swarm intelligence optimization methods have garnered considerable attention due to their exceptional global search capabilities. Among these, BSO, an emerging heuristic optimization framework, has demonstrated promising exploration performance and adaptability [12,13]. Standard BSO algorithms employ K-Means as the core clustering technique to guide individual partitioning and new solution generation during the search process. However, K-Means itself is sensitive to initial centroids, exhibits poor robustness to outliers, and necessitates a pre-defined number of clusters. In high-dimensional complex problems, such as multi-robot task allocation, these limitations often lead to uneven partitioning of the search space and distorted cluster structures, thereby compromising the algorithm’s stability and global performance [14,15].

Meanwhile, although traditional clustering methods like K-Means and DBSCAN are widely applied in task clustering and allocation, a singular clustering strategy often struggles to concurrently ensure both clustering efficiency and robustness. Particularly in environments with dense or irregularly shaped task distributions, these methods are prone to issues such as cluster drift and indistinct boundaries, thereby limiting their effectiveness in complex multi-robot scenarios [14,16].

To address the aforementioned issues, this paper proposes an HC-BSO algorithm, which is designed to improve both the solution efficiency and path quality in MRPP. Our approach synergizes the rapid clustering capabilities of Mini-Batch K-Means with the density-based identification and noise robustness inherent to DBSCAN. This fusion allows us to construct a multi-level task clustering mechanism, thereby enhancing the structural representation capabilities of the swarm intelligence algorithm [17,18,19]. Building on this, HC-BSO incorporates dynamic population initialization to bolster search diversity. Additionally, we have designed an exploration–perturbation dual-stage evolutionary mechanism to enhance the search efficiency and precision for both global and local solutions.

The primary contributions of this research are summarized as follows:

- We propose a hybrid clustering technique that integrates Mini-Batch K-Means and DBSCAN to bolster the clustering efficiency and robustness of the BSO algorithm in multi-robot task allocation.
- We designed a dynamic population initialization mechanism and a dual-stage evolutionary strategy to enhance the algorithm’s global exploration and local optimization capabilities during the path search process.
- Through systematic simulations, we demonstrate that HC-BSO outperforms several mainstream swarm intelligence algorithms in terms of computation time, path length, and path conflict avoidance.

The remainder of this paper is organized as follows:

Section 2 introduces the related research status of multi-robot path planning.

Section 3 reviews the basic principles of the BSO algorithm and mainstream clustering techniques.

Section 4 describes in detail the proposed HC-BSO algorithm and its key components, including task modeling, hybrid clustering, and path planning mechanisms.

Section 5 presents the simulation experiment setup and results, and systematically analyzes the various performance indicators.

Section 6 discusses the advantages and limitations of the algorithm.

Finally, Section 7 summarizes the full text and outlines future research directions.

## 2. Related Work

MRPP, a critical technology in intelligent robotic systems, has seen the emergence of diverse research avenues in recent years. These can be broadly categorized into four main types: exact algorithms, sampling-based methods, reactive approaches, and swarm intelligence optimization algorithms, the last of which have garnered significant attention recently [20,21,22].

Traditional path planning methods were predominant in early MRPP research. Among these, exact algorithms such as A* and Dijkstra can guarantee globally optimal path solutions; however, their high computational complexity typically limits their applicability to small-scale or single-robot path planning problems [23,24,25,26]. To tackle path search in high-dimensional spaces, sampling-based methods like Rapidly Exploring Random Trees (RRT) have found widespread application. While these methods offer advantages in terms of path diversity, they may still exhibit shortcomings regarding path quality and stability [27,28]. Furthermore, reactive approaches, for instance, the Dynamic Window Approach (DWA), demonstrate good adaptability in dynamic environments owing to their real-time responsiveness and straightforward implementation. Yet, their global planning capabilities are somewhat limited, often leading them to become trapped in local optima. Zhou et al. [29] enhanced DWA performance for single-robot navigation by integrating several improvement strategies. Extending this method to multi-robot systems, however, introduces considerable challenges related to path conflict resolution and collaborative control.

To overcome the limitations of traditional methods, swarm intelligence optimization algorithms have become a mainstream research direction in MRPP in recent years. This is largely due to their robust global search capabilities, adaptive parameter tuning mechanisms, and aptitude for modeling complex problems. Prominent examples include Genetic Algorithms (GA) [30,31], Simulated Annealing (SA) [32], Ant Colony Optimization (ACO) [33,34], Particle Swarm Optimization (PSO) [35], Firefly Algorithm (FA) [36,37], Whale Optimization Algorithm (WOA) [38,39], and Grey Wolf Optimizer (GWO) [40,41]. For instance, Cai et al. [42] employed FA to optimize parameters within ACO for multi-robot collaborative path generation, achieving commendable path quality enhancements, though computational efficiency could be further optimized. Yang et al. [33] proposed a multi-robot path planning method founded on an improved Ant Colony Algorithm (LF-ACO) and a leader-follower strategy, which effectively realized formation keeping and obstacle avoidance in complex environments. Pu et al. [34] improved ACO using a gradient pheromone initialization strategy, leading to superior performance in multi-objective path planning tasks; nevertheless, their approach still faces challenges concerning system scalability and path conflict resolution. The Strength Learning Particle Swarm Optimization (SLPSO) algorithm, introduced by Liu et al. [35], integrates strength learning and local search mechanisms to enhance robustness and task completion efficiency in large-scale task scheduling, yet it grapples with the common issue of premature convergence. You et al. [39] developed the improved WOA (IWOA), incorporating an improved tent chaotic map and an adaptive inertia strategy, which effectively boosted search capability and convergence speed, particularly for small-scale multi-robot systems. Tian et al. [43] fused WOA and FA to create Firefly with Whale Optimization Algorithm (FWOA), enhancing the algorithm’s exploitation and exploration capabilities through a multi-population opposition-based learning mechanism for function optimization and path search tasks. Dong et al. [44], in a different vein, proposed an improved GWO (IGWO) by introducing a dynamic local optimum escape strategy and an individual repositioning mechanism, significantly enhancing its global search ability and path quality. While these methods demonstrate strong performance in areas like path planning and task scheduling, they often present limitations in managing path conflict avoidance and collaborative control for large-scale multi-robot systems. Furthermore, many of these algorithms still offer scope for improvement concerning convergence speed, computational efficiency, or path length optimization.

In multi-robot path planning, task partitioning strategies critically influence overall planning effectiveness. Consequently, recent research has increasingly investigated the integration of clustering methods to assist in task allocation, aiming to enhance partitioning efficiency and alleviate the subsequent path search burden. For instance, Yuan et al. [45] proposed a combination of an improved K-Means++ and a PSO algorithm for task point clustering and sorting optimization. This approach yielded significant improvements in task allocation efficiency and control over total path length, though it did not fully address path conflict issues. Liu et al. [46] combined equal-sized K-Means (ESCA) with an asynchronous genetic algorithm (AGA) for task partitioning and path sub-optimization, thereby enhancing scheduling efficiency in large-scale task scenarios. However, this work did not effectively integrate practical factors such as robot workload and path conflicts. The Adaptive Memory Algorithm (AMA) introduced by Cheng et al. [47] and the Simulated Annealing-based job scheduling approach (SJSA) by Huo et al. [32], respectively, simplified the multi-robot path planning problem through a two-layer local search mechanism and virtual node modeling. While these methods demonstrated advantages in path length control and load balancing, they have yet to thoroughly resolve issues concerning path conflicts and robustness, particularly in task-dense environments.

Clustering mechanisms have also found widespread integration within swarm intelligence optimization frameworks, where they assume a pivotal role, especially in BSO. BSO leverages clustering strategies to group individuals, guiding the search direction; standard K-Means clustering is commonly employed as its foundational mechanism. However, K-Means exhibits limitations when confronted with high-density, non-convex shapes, noise interference, or dynamic task distributions. These include sensitivity to initial cluster centroids, poor robustness to outliers, and a strong dependency on the pre-specified number of clusters [15,48,49]. These shortcomings mean that traditional BSO, when applied to multi-robot task partitioning and path planning, often suffers from unstable clustering quality, significant computational overhead, and diminished capacity to maintain population diversity. Such issues consequently limit its practical utility in large-scale, high-density path optimization scenarios.

While existing multi-robot path planning methods have achieved considerable progress in optimization performance, search capabilities, and task allocation, several prominent issues persist: (1) Clustering processes often lack sufficient adaptability and stability, struggling to cope effectively with dynamic task densities and irregular distributions. (2) Swarm intelligence optimization methods are not yet fully mature concerning path conflict avoidance and scalability in multi-robot systems. (3) Current research generally lacks mechanisms for a deep, synergistic integration of clustering processes with intelligent search procedures.

To address these challenges, this paper introduces a HC-BSO algorithm. Our approach improves clustering quality and task partitioning robustness by fusing Mini-Batch K-Means with the density-based clustering algorithm DBSCAN. Furthermore, HC-BSO incorporates an exploration-perturbation evolutionary mechanism to bolster search capabilities, aiming to achieve an optimal balance among path quality, conflict avoidance, and computational efficiency in large-scale multi-robot task scenarios.

## 3. BSO Algorithm and Mainstream Clustering Techniques

### 3.1. BSO Algorithm

The BSO is a population-based optimization technique inspired by the human brainstorming process. The central idea mimics a group of individuals engaging in collective discussion to identify and refine the best possible solutions. In this algorithm, each candidate solution is treated as an “idea”, which evolves iteratively through a brainstorming-like mechanism. Specifically, the BSO algorithm clusters these ideas into several groups, within which local optimization is performed to identify a local optimum. The best solutions from all clusters are then compared to determine the global optimum. To mitigate the risk of premature convergence to local minima, BSO incorporates mutation operations that introduce random perturbations, thereby maintaining solution diversity throughout the search process. This mechanism enables BSO to efficiently locate the global optimum, even for complex optimization problems [50]. This capability is particularly critical in practical domains like multi-robot path planning, where time efficiency directly impacts overall system performance. Furthermore, the algorithm is characterized by a simple structure, a small number of parameters, and ease of implementation.

In the context of BSO, the optimization objective is typically defined by a cost function J(p), where *p* represents the set of path points corresponding to a candidate solution in robot path planning. The goal is to search for a solution that minimizes this cost function, formally expressed as follows:

(1) $$\min_{p}J\left(p\right).$$

Clustering plays a pivotal role in the local optimization phase of the BSO algorithm. The *k*-means clustering algorithm is employed to group the solution space into clusters, within which each solution is optimized independently. The optimal solution from each cluster is then compared to identify the globally best solution. Mathematically, the optimization within each cluster can be expressed as follows:

(2) $$\min_{p\in C_{i}}f\left(p,c_{i}\right).$$

where $C_{i}$ denotes the *i*-th cluster, $c_{i}$ represents the centroid of that cluster, and $f(p,c_{i})$ is the cost function defined within the cluster. The intra-cluster cost function $f(p,c_{i})$ is defined as the squared Euclidean distance between point *p* and its corresponding cluster center $c_{i}$, i.e., $f\left(p,c_{i}\right)=||p-c_{i}{\left|\right|}^{2}$. The objective of intra-cluster optimization is to minimize the cumulative distance from each point to its corresponding cluster center, thereby achieving local path optimization within each cluster. This objective is mathematically aligned with the minimization of the within-cluster sum of squares (WSS) criterion used in K-Means clustering. Formally, WSS can be expressed as follows:

(3) $$\mathrm{WSS}=\sum_{i=1}^{k}\sum_{p_{j}\in C_{i}}\left|\right|p_{j}-c_{i}{\left|\right|}^{2}.$$

In this context, intra-cluster optimization can be regarded as a subproblem of the overall optimization objective (see Equation (1)). By minimizing $f(p,c_{i})$ within each cluster, BSO algorithm iteratively approaches the global minimum of the overall cost function J(p).

To prevent the algorithm from becoming trapped in local optima, a mutation mechanism is incorporated into the BSO framework. This mechanism consists of two types of mutation: intra-cluster mutation and inter-cluster fusion mutation. The intra-cluster mutation introduces controlled random perturbations to the current solution *p* in order to generate a new candidate solution $p^{\prime }$. This process can be described as

(4) $$p^{\prime }=p+\eta ,$$

where η is the perturbation parameter. This perturbation can take the form of Gaussian noise or other random variations, aimed at exploring new directions in the search space and enhancing the algorithm’s ability to escape local minima.

Inter-cluster recombination, often referred to as crossover, is designed to generate new individuals by combining features from two or more existing solutions. In BSO, this is achieved by integrating individuals or centroids from different clusters. This strategy allows the algorithm to merge information from multiple locally optimal regions, thereby producing new candidates with potentially superior fitness. Such recombination not only promotes population diversity but also facilitates the search beyond local optima, increasing the likelihood of finding the global best solution [51,52].

Together, intra-cluster mutation and inter-cluster recombination constitute the primary mechanisms for generating new individuals in BSO. Intra-cluster mutation maintains diversity within each cluster by introducing randomness at the level of individuals or cluster centers. In contrast, inter-cluster recombination enables cross-cluster knowledge sharing and hybridization. The synergy between these two operations strikes a balance between exploration and exploitation, enhancing both the local search efficiency and global search capability of the algorithm. As a result, BSO demonstrates strong performance in solving complex optimization problems by effectively navigating the trade-off between convergence speed and solution quality.

### 3.2. Mainstream Clustering Techniques

In MRPP, clustering techniques play a crucial role by partitioning task points into manageable subsets. This partitioning facilitates the assignment of specific task regions to individual robots, thereby reducing the overall problem complexity and enhancing collaborative efficiency. As an integral component of the BSO algorithm, the clustering operation directly impacts both the rationality of task allocation and the precision of subsequent path optimization. This subsection briefly analyzes the characteristics of five clustering techniques—K-Means, Mini-Batch K-Means, DBSCAN, Agglomerative Clustering (AC), and Gaussian Mixture Models (GMMs)—and assesses their suitability for MRPP applications [15,16,17,53,54]. These aspects are summarized in Table 1.

While each of the aforementioned clustering techniques offers distinct advantages, a singular approach often struggles to concurrently meet the comprehensive requirements of MRPP, which demands a balance of efficiency, precision, and robustness. For example, K-Means and Mini-Batch K-Means are computationally efficient but can be sensitive to noise and outliers. DBSCAN, on the other hand, is adept at handling complex, arbitrarily shaped clusters and is robust to noise, yet it may incur significant computational overhead, particularly with large datasets. AC and GMM provide flexibility in cluster definition; however, they generally exhibit lower computational efficiency compared to simpler methods like K-Means. Consequently, to optimize the BSO algorithm’s performance in MRPP, it becomes apparent that hybrid clustering strategies, designed to synergistically combine the strengths of multiple techniques, are necessary.

## 4. Methodology

### 4.1. Problem Formulation

The MRPP problem addresses the challenge of determining optimal paths for *k* robots. These paths originate from the robots’ respective starting locations, require them to visit a subset or all points within a designated task set $X=\{p_{1},p_{2},\ldots ,p_{n}\}$, and culminate in their return to these initial starting points. In this work, we decompose this problem into two primary stages: task allocation and path planning. The task allocation stage partitions the entire set of task points *X* into *k* disjoint subsets, with each subset being assigned to a specific robot. Subsequently, the path planning stage focuses on computing an optimal route for each robot to traverse all tasks within its allocated subset.

Let the coordinates of a task point $p_{i}$ be denoted by $\left(x_{i},y_{i}\right)$, and let $s_{r}$ represent the starting location of robot *r*. The distance $d(p_{i},p_{j})$ between any two points $p_{i}$ and $p_{j}$ is typically computed using the Euclidean distance:

(5) $$d\left(p_{i},p_{j}\right)=\sqrt{{\left(x_{i}-x_{j}\right)}^{2}+{\left(y_{i}-y_{j}\right)}^{2}}.$$

For a robot *r*, its assigned subset of task points is denoted as $X_{r}\subseteq X$. The path for robot *r*, designated $P_{r}$, is defined as a sequence commencing at $s_{r}$, visiting every point in $X_{r}$ precisely once, and concluding by returning to $s_{r}$. We can represent this path as the sequence $P_{r}=\left(s_{r},v_{r,1},v_{r,2},\ldots ,v_{r,|X_{r}|},s_{r}\right)$, where the set $\left\{v_{r,1},v_{r,2},\ldots ,v_{r,|X_{r}|}\right\}$ constitutes a specific permutation of the points within $X_{r}$. The total length of robot *r*’s path, $L(P_{r})$, is then the sum of the distances between consecutive points along this sequence:

(6) $$L\left(P_{r}\right)=d\left(s_{r},v_{r,1}\right)+\sum_{i=1}^{|X_{r}|-1}d\left(v_{r,i},v_{r,i+1}\right)+d\left(v_{r,|X_{r}|},s_{r}\right).$$

The overarching objective in MRPP is to identify a task assignment scheme, represented by $\left\{X_{1},X_{2},\ldots ,X_{k}\right\}$, and a corresponding set of paths $\left\{P_{1},P_{2},\ldots ,P_{k}\right\}$. This scheme aims to either minimize the maximum path length among all robots (often referred to as the min-max objective) or minimize the sum of the path lengths of all robots. The specific objective of this study is to minimize the sum of the total path lengths for all robots:

(7) $$\min\sum_{r=1}^{k}L\left(P_{r}\right).$$

This minimization is subject to the following constraints:

(8) $$\overset{k}{\underset{r=1}{⋃}}X_{r}=X,\;X_{i}\cap X_{j}=\emptyset \;\forall i\neq j.$$

Furthermore, each robot *r* is required to visit every task point within its assigned subset $X_{r}$ exactly once.

### 4.2. Hybrid Clustering-Based Task Allocation

Task allocation is an initial and critical step in MRPP, involving the systematic assignment of a set of task points, denoted as *X*, to *k* available robots. Conventional clustering algorithms, such as K-Means, exhibit sensitivity to initial centroid placement and are prone to converging to local optima. Conversely, while DBSCAN can identify clusters of arbitrary shapes, its parameters can be challenging to tune, and it is susceptible to noise [15,16]. Recognizing that task point distributions in practical applications are often irregular and may contain noise, this paper proposes a hybrid clustering strategy that integrates Mini-Batch K-Means with DBSCAN for task allocation. The rationale is that Mini-Batch K-Means can rapidly process large-scale datasets and mitigate the dependency on initial centroid selection, while DBSCAN can subsequently refine cluster boundaries and identify outliers.

The specific procedure for this hybrid clustering strategy is described as follows.

Step 1: Mini-Batch K-Means is first employed for an initial clustering of the task point set *X*. The objective is the minimization of the within-cluster sum of squares (WSS) (Equation  (3)). By iteratively processing mini-batches of size *b*, this algorithm rapidly generates an initial set of *k* clusters, $\left\{C_{1},C_{2},\ldots ,C_{k}\right\}$, along with their respective centroids $c_{1},c_{2},\ldots ,c_{k}$. Mini-Batch K-Means updates centroids by iteratively sampling mini-batches of data; the centroid update rule is

(9) $$c_{i}^{\left(t+1\right)}=c_{i}^{\left(t\right)}+\eta \cdot \frac{\sum_{p_{j}\in B_{i}}\left(p_{j}-c_{i}^{\left(t\right)}\right)}{|B_{i}|}.$$

where $c_{i}^{\left(t\right)}$ represents the centroid of the *i*-th cluster at iteration *t*, $B_{i}$ is the set of samples within the current mini-batch assigned to cluster *i*, $|B_{i}|$ denotes the cardinality of $B_{i}$ (i.e., its size), and η is the learning rate. This initial step efficiently generates *k* approximate task point groupings.

Step 2: For each preliminary cluster $C_{i}$ generated by Mini-Batch K-Means, the DBSCAN algorithm is subsequently applied to perform further density analysis and refinement. DBSCAN defines clusters based on point density, characterized by two parameters: the neighborhood radius ϵ and the minimum number of points, $min_{points}$, required to establish a core point. For an arbitrary point $p_{j}\in C_{i}$, its ϵ-neighborhood, $N_{\epsilon }\left(p_{j}\right)$, is defined as the set of points within $C_{i}$ whose distance to $p_{j}$ is less than or equal to ϵ:

(10) $$N_{\epsilon }\left(p_{j}\right)=\left\{p_{l}\in C_{i}|d\left(p_{j},p_{l}\right)\leq \epsilon \right\}.$$

If the cardinality of this neighborhood, $|N_{\epsilon }\left(p_{j}\right)|$, meets or exceeds $min_{points}$, then $p_{j}$ is identified as a core point. DBSCAN leverages these core points and their density-reachable counterparts to discover clusters of arbitrary shapes. Points that do not qualify as core or boundary points are designated as noise points (labeled − 1).

Step 3: Following the DBSCAN process, some points may be classified as noise, indicating they were not assimilated into any valid density-based cluster. To ensure comprehensive task allocation to the robots, these noise points necessitate reassignment. Specifically, for any noise point $p_{j}$ (identified with a label of −1 by DBSCAN), we reassign it to the cluster associated with the Mini-Batch K-Means centroid closest to $p_{j}$. This reassignment is governed by the following rule.

(11) $$l_{j}=\arg\min_{1\leq i\leq k}d\left(p_{j},c_{i}\right),$$

where $c_{i}$ represents the centroid of the *i*-th cluster derived from the initial Mini-Batch K-Means clustering phase. This procedure guarantees that all task points are incorporated into the subsequent path planning stages.

Through the aforementioned hybrid clustering procedure, the set of task points *X* is effectively partitioned into *k* mutually exclusive subsets: $X_{1},X_{2},\ldots ,X_{k}$. Subsequently, each subset $X_{r}$ is assigned to a corresponding robot *r*. Each robot is then tasked with planning the shortest path to visit all points within its assigned subset $X_{r}$.

### 4.3. Path Planning

Following the assignment of its task point set $X_{r}$, each robot *r* must plan a shortest path that visits all its assigned points and subsequently returns to its origin. This subproblem constitutes a variant of the classic Traveling Salesman Problem (TSP). Given the NP-hard nature of the TSP, which renders exact solutions computationally prohibitive for larger instances, this work employs an improved BSO algorithm. BSO is a metaheuristic algorithm that emulates the collective problem-solving process of human groups; we have adapted and enhanced this algorithm specifically for the path planning context.

#### 4.3.1. Population Initialization

For a given robot *r*, let its assigned set of task points be $X_{r}$. We assume this set contains $n_{r}=\left|X_{r}\right|$ task points. A solution, in this context, represents a specific sequence in which robot *r* visits these $n_{r}$ task points. We encode a solution as a permutation of task point indices. Specifically, a solution is represented by a sequence $S=(s_{1},s_{2},\ldots ,s_{n_{r}})$, where $\left(s_{1},s_{2},\ldots ,s_{n_{r}}\right)$ is a permutation of the set $\left\{1,2,\ldots ,n_{r}\right\}$. This permutation dictates the robot’s trajectory: starting from its initial location, the robot first visits the task point in $X_{r}$ corresponding to index $s_{1}$, then proceeds to the task point with index $s_{2}$, and so on, until it has visited the task point indexed by $s_{n_{r}}$. Finally, the robot returns to its starting point.

The complete path encompasses the starting point, the task points visited in sequence *S*, and a final return to the starting point. The total length of this path serves as the metric for evaluating the solution’s quality.

Before the execution of the improved BSO algorithm, an initial population is constructed for each robot *r*. This population, denoted $P_{r0}$, comprises a set of *m* independent solutions. These initial solutions represent *m* different permutations for visiting the assigned task points, thereby forming the starting points for the algorithm’s search for an optimal path.

To ensure broad coverage of the initial search space and maintain population diversity, we generate each solution within the initial population $P_{r0}$ by creating a uniformly random permutation of the $n_{r}$ task point indices $\left\{1,2,\ldots ,n_{r}\right\}$ assigned to robot *r*. In other words, for each solution $S_{i}\in P_{r0}$ (where i=1,…,m), it represents a randomly generated permutation of these $n_{r}$ indices.

(12) $$P_{r0}=\left\{S_{1},S_{2},\ldots ,S_{m}\right\}.$$

where each $S_{i}=\left(s_{i,1},s_{i,2},\ldots ,s_{i,n_{r}}\right)$ is a random permutation of $\left\{1,2,\ldots ,n_{r}\right\}$.

This random initialization strategy is instrumental in preventing premature convergence to local optima and provides a diverse foundation for subsequent exploration and perturbation operations. Once initialization is complete, the path length (i.e., fitness value) for each solution in the population is calculated, in preparation for the ensuing optimization iterations.

#### 4.3.2. Enhanced BSO Operations

To effectively tackle the complex optimization challenge inherent in multi-robot path planning, we have adapted the standard BSO algorithm. Our enhancements center on a dynamic evolutionary mechanism specifically tailored for multi-robot scenarios. This mechanism leverages a dual evolutionary strategy, encompassing both exploration and perturbation. We found this combined approach to significantly improve solution diversity, convergence speed, and the overall quality of the planned paths. The exploration phase broadens the global search by combining disparate candidate solutions, thereby aiming to uncover novel, potentially superior paths. Perturbation, conversely, focuses on refining path quality within the local neighborhood of a current solution through targeted adjustments to its visitation sequence. Together, these two components form the core of our dual evolutionary strategy, striking a crucial balance between global exploration and local exploitation capabilities.

Exploration Operation: The exploration operation aims to emulate the combination of beneficial traits from existing solutions (parents) to generate new solutions (offspring), thereby probing unexplored regions of the solution space. Specifically, we implement an order-based crossover mechanism [55]. Two-parent solutions $P_{a}$ and $P_{b}$ are selected from the population (e.g., chosen randomly from the same cluster or selected as the cluster’s representative solutions). A random crossover point $c\in [1,n_{r}-1]$ is determined, where $n_{r}$ denotes the total number of task points. The generation of an offspring solution $P^{\prime }$ proceeds as follows:

The initial segment of $P_{a}$, comprising the first *c* task points, is directly copied to the corresponding positions in the offspring $P^{\prime }$:

(13) $$P^{\prime }\left(1:c\right)=P_{a}\left(1:c\right).$$

Subsequently, task points from the parent $P_{b}$ that are already present $P^{\prime }\left(1:c\right)$ are identified and excluded from contributing to the offspring.

The remaining task points from $P_{b}$ are then appended to $P^{\prime }$ in their original relative order from $P_{b}$, populating the vacant positions in $P^{\prime }$.

This methodology preserves partial structural information inherited from the parents while simultaneously generating novel sequences through the amalgamation of segments from disparate parental solutions. Such a process facilitates the creation of offspring that can exhibit considerable divergence from their progenitors, potentially leading to superior solutions.

Perturbation Operation: This operation, analogous to individual brainstorming or fine-tuning ideas, generates new solutions by introducing local modifications to a single existing solution. Its primary aims are to enhance population diversity and to mitigate the risk of premature convergence to local optima [56]. We implement this using a swap mutation operator. For a selected parent solution *P*, two distinct position indices $j_{1},j_{2}\in \left[1,n_{r}\right]$ (where $j_{1}\neq j_{2}$), are randomly chosen. The task points at these two positions are then exchanged to create a new offspring solution $P^{\prime }$. This can be represented mathematically as:

(14) $$P^{\prime }=\mathrm{swap}\left(P,j_{1},j_{2}\right),\;j_{1}\neq j_{2},$$

where $\mathrm{swap}(P,j_{1},j_{2})$ signifies the operation of exchanging the $j_{1}$-th and $j_{2}$-th elements within solution *P* to yield the new solution $P^{\prime }$.

In each iteration of the algorithm, every candidate solution selects either the exploration or the perturbation operation with a probability of 0.5. This dual evolutionary strategy, leveraging the synergistic effects of exploration and perturbation, demonstrably enhances the algorithm’s adaptability within complex MRPP scenarios, offering a more robust approach compared to the singular update mechanism found in the standard BSO.

Subsequent to the generation of new solutions (via either exploration or perturbation), the population is updated by a process of cost evaluation and selection, which also involves tracking the best-performing solution found so far. The cost of a candidate solution is quantified by its total path length, specifically calculated using a ‘route()’ function. If we define *p* as a complete path—comprising the robot’s starting point (origin), the $n_{r}$ assigned task points in sequence, and the return to the origin—the total distance *D* is calculated as follows:

(15) $$D=\sum_{i=1}^{n_{r}+1}{∥p_{i+1}-p_{i}∥}_{2}.$$

In this formulation, $p_{i}$ represents the coordinates of the *i*-th point along the path (where $p_{1}$ is the origin and $p_{n_{r}+2}$ is the return to the origin after visiting all $n_{r}$ task points $p_{2},\ldots ,p_{n_{r}+1}$), ${∥\cdot ∥}_{2}$ denotes the Euclidean norm, and *D* is the total path distance. If the calculated cost *D* of a newly generated solution is lower than that of its parent or the solution it is intended to replace, the new solution takes its place in the population. Furthermore, if this new path’s cost is lower than the current global best cost, the “best path” and “best length” records are updated. This selection mechanism ensures that the population progressively evolves towards solutions of lower cost.

To monitor the optimization progress, convergence is tracked by generating a convergence curve, which is computed as the average of the optimal path lengths for all robots. At the *t*-th iteration,

(16) $$V\left(t\right)=\frac{1}{k}\sum_{r=1}^{k}B_{r}.$$

Here, V(t) represents the value of the convergence curve at iteration *t*, *k* is the total number of robots in the system, and $B_{r}$ denotes the optimal path length found by the *r*-th robot at the current iteration. The convergence curve not only reflects the algorithm’s convergence trend but also facilitates performance comparisons with other methodologies. In contrast to standard BSO, which often lacks a dynamically adjusted optimization process, the iterative evolution in our proposed method introduces new candidate solutions in each iteration through exploration and perturbation mechanisms, while simultaneously preserving high-quality solutions. This dual approach ensures both the efficiency and stability of the search process.

### 4.4. Algorithmic Procedure and Parameter Settings

Based on the above steps, a BSO algorithm enhanced by hybrid clustering is proposed for multi-robot path planning. The corresponding Algorithm 1 code is as follows.

To ensure the proposed HC-BSO algorithm achieves optimal performance in multi-robot path planning scenarios and to enhance the reliability of our experimental results, we systematically identified and tuned its key parameters. These parameters primarily govern the core components of the HC-BSO algorithm, encompassing optimization parameters for the foundational BSO, clustering parameters for the hybrid clustering process, and control parameters for the dual evolutionary strategies. Specifically, the critical parameters requiring tuning include population size (pop_size), maximum number of iterations (max_iter), batch size for Mini-Batch K-Means (batch_size), neighborhood radius (ϵ) and minimum number of points to form a core object (min_pts) for DBSCAN, and the probabilities for the exploration and perturbation strategies ($P_{explore}$ and $P_{perturb}$, respectively).


**Algorithm 1:** Multi-robot path planning via Hybrid Clustering and BSO**Require:** Target points $P=\{p_{1},\ldots ,p_{N}\}$, center point *C*, number of robots *k*, BSO       parameters: pop_size, max_iter**Ensure:** Optimized robot paths $\left\{S_{1},\ldots ,S_{k}\right\}$ and total distance *D*
  1:**Initialization:** Load coordinates *P*; set center C=(0.5,0.5)  2:
**Step 1: Hybrid Clustering for Task Assignment**
  3:Apply Mini-Batch K-Means on *P* to obtain initial clusters $\left\{C_{1},...,C_{k}\right\}$ and centroids  4:Refine clusters using DBSCAN to detect and label noise points  5:Assign noise points to nearest centroids based on Euclidean distance  6:**for** each robot r=1 to *k* **do**  7:    Assign cluster $C_{r}$ as robot *r*’s task set $X_{r}$  8:    **Step 2: Enhanced BSO Operations for Path planning**  9:    Create population $Pop_{r}=\left\{S_{1},...,S_{m}\right\}$ of *m* permutations over $X_{r}$10:  Set best cost $B_{r}\leftarrow \infty$11:  **for** iteration t=1 to max_iter **do**12:      **for** each $S\in Pop_{r}$ **do**13:         **if** rand <0.5 **then**14:             *Exploration:* generate child via sequence crossover15:         **else**16:             *Perturbation:* generate child via random index swap17:         **end if**18:      **end for**19:      Evaluate path cost D(S) using $D=\sum_{i=1}^{n+1}{∥p_{i+1}-p_{i}∥}_{2}$20:      Update $Pop_{r}$ by replacing worse individuals21:      Update global best $B_{r}$ and path $S_{r}$ if better child found22:  **end for**23:  Store $S_{r}$ and $B_{r}$ for robot *r*24:
**end for**
25:**Output:** Total distance $D=\sum_{r=1}^{k}B_{r}$, and all optimized robot paths $\left\{S_{r}\right\}$



The parameter tuning process was conducted using the One-Factor-at-a-Time (OFAT) method [57,58]. These tuning experiments were performed within a representative simulation scenario involving 30 task points and 5 robots. During this process, only one parameter’s value was varied at a time, while all other parameters were maintained at their baseline or previously optimized values. For each parameter configuration, the algorithm was executed independently 10 times. We utilized a weighted combination of total path length and computation time as the primary performance evaluation metric, recording both the mean and standard deviation to comprehensively assess solution quality and algorithmic stability. The search ranges for each parameter were established as follows: pop_size∈{20,30,50,80}; max_iter∈{100,200,300,500}; batch_size∈{10,20,30,50}; ϵ∈{5,8,10,15}; min_pts∈{1,2,3,4}. The ratio of exploration probability to perturbation probability was selected from three combinations: {0.3/0.7,0.5/0.5,0.7/0.3}.

Based on this systematic tuning experimentation and subsequent analysis, we identified a parameter configuration that demonstrated robust overall performance across various test scenarios. This set of parameters was then consistently applied in all subsequent comparative experiments. The selected parameter values are pop_size=30, max_iter=300, batch_size=20, ϵ=10, min_pts=2, with both the exploration probability ($P_{explore}$) and perturbation probability ($P_{perturb}$) set to 0.5. This combination strikes an effective balance between solution accuracy and computational efficiency, exhibiting favorable performance in terms of total path length, run time, convergence characteristics, and stability, thereby ensuring the reliability of the algorithm and the reproducibility of the experimental findings.

## 5. Experiments and Results

This chapter presents simulation experiments designed to evaluate the performance of different clustering techniques within the framework of BSO for MRPP and to validate the efficacy of our proposed hybrid clustering method, HC-BSO. Our experimental procedure is structured in three main phases: First, we compare the performance of five conventional clustering techniques—K-Means, Mini-Batch K-Means, DBSCAN, AC, and GMM—against our HC-BSO algorithm. Second, we evaluate the capability of HC-BSO to prevent path intersections, highlighting its potential advantages. Finally, a comprehensive comparison is drawn between HC-BSO and other established swarm intelligence algorithms, namely FA, SA, GWO, ACO, and WOA. The results are quantitatively assessed based on metrics including computational time, total path length, and the ability to mitigate path conflicts.

### 5.1. Experimental Setup

**Figure 1:**
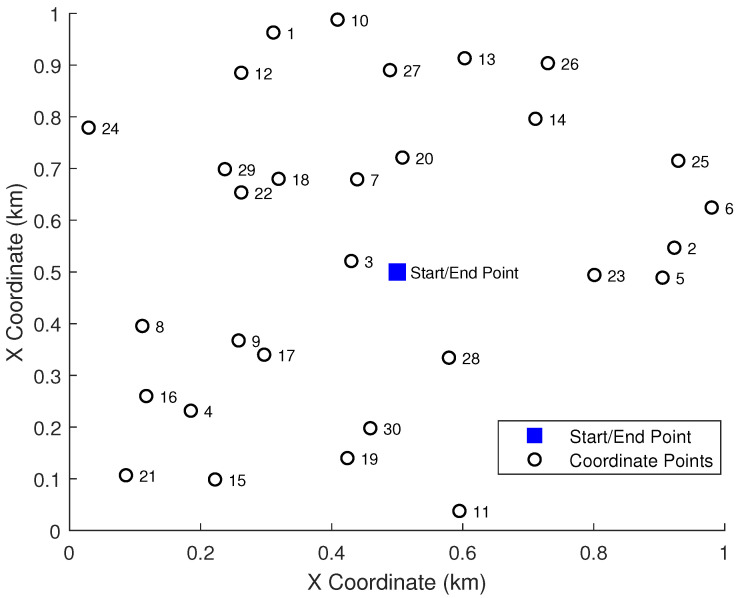
Simulation map depicting 30 randomly distributed task points.

In this work, we conducted a series of experiments to assess the performance of various clustering techniques when integrated into the BSO algorithm for MRPP. The experimental environment was a 1km×1km unobstructed, open area, within which 30 task points were randomly distributed. Five robots initiated their paths from a common depot at (0.5km,0.5km) and were required to return to this depot after servicing their assigned tasks. The simulation map is depicted in Figure 1.

Experiments were conducted using MATLAB R2021b on a system equipped with an AMD Ryzen 7 6800H (3.20 GHz) processor, running a 64-bit Windows 11 operating system. To ensure the statistical reliability of our findings, each method underwent 20 independent experimental runs, with each run comprising 300 iterations. A fixed random seed was employed across all executions to guarantee reproducibility. We meticulously recorded the computational time for each experiment and the total path distance achieved after 300 iterations; these two metrics served as the primary criteria for assessing the performance of different clustering techniques.

### 5.2. Validation of Clustering Improvement

In MRPP, task assignment and path planning are intrinsically coupled. Clustering algorithms, by partitioning task points among robots, effectively define the initial conditions for the subsequent path optimization phase, thereby directly impacting the total path length. This section aims to validate the comprehensive influence of our improved hybrid clustering method, HC-BSO, on both task assignment efficiency and the quality of the resultant paths. This is achieved through a comparative performance analysis against five conventional clustering algorithms: K-Means, Mini-Batch K-Means, DBSCAN, AC, and GMM. The experimental settings, as detailed in Section 4.1, were maintained for this validation. Each clustering approach was subjected to 20 independent trials, with each trial encompassing 300 iterations.

Table 2 presents the performance of six clustering algorithms within the BSO framework, detailing average computation times and average total path lengths derived from 20 experimental runs. Results are presented with 95% Confidence Intervals (CI) to mitigate the influence of stochasticity and enhance statistical validity.

As evident from Table 2, the integration of different clustering techniques with the BSO framework yields significant variations in their performance characteristics. Among the traditional clustering methods, K-Means and Mini-Batch K-Means demonstrated relatively fast computation times, averaging 75.79 s and 76.93 s, respectively. However, K-Means’ sensitivity to initial centroid selection resulted in the longest average total path length (7.84 km) and substantial fluctuations, indicated by its wide confidence interval. Mini-Batch K-Means, by updating centroids using subsets of the data, maintained rapid computation while achieving the lowest average total path length (7.01 km) among these traditional approaches, thereby highlighting its potential for path optimization. Although DBSCAN exhibited a slightly longer computation time (80.82 s), its density-based clustering nature endowed it with robustness in handling non-spherical or noisy data distributions, enabling it to achieve the second-lowest average total path length (7.16 km). The performance of Hierarchical Clustering and GMM was intermediate to these aforementioned methods.

Notably, our proposed HC-BSO method significantly outperforms the aforementioned individual clustering techniques across all key metrics. It achieved an average computation time of just 0.77 s, which is a striking improvement of approximately 98.98% in computational efficiency compared to the 75.79 s required by K-Means. Regarding path length, our improved method yielded an optimal average total path length of 6.42 km. This represents an 18.08% reduction from K-Means’ 7.84 km and also surpasses the best results obtained by Mini-Batch K-Means and DBSCAN. Crucially, the HC-BSO method exhibits exceptionally narrow 95% CI (time: [0.744, 0.796] s; path length: [6.390, 6.448] km). These are considerably tighter than those associated with the five traditional methods, indicating very low volatility, stable performance, and a high degree of reproducibility in our experimental results.

**Figure 2:**
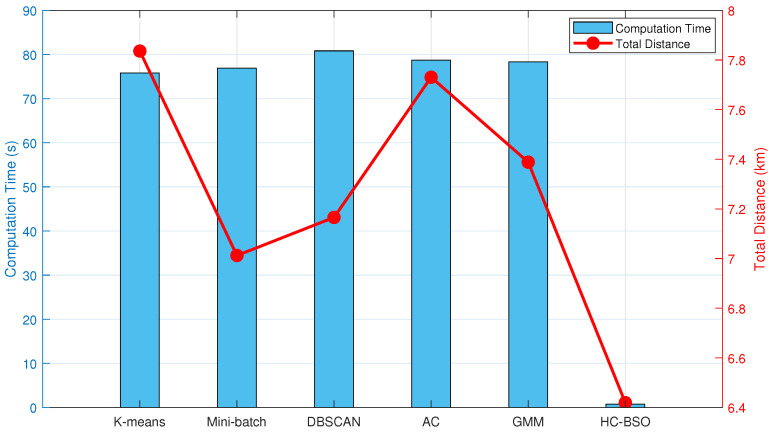
Dual-axis plot of computation time versus total distance for six clustering techniques.

The performance statistics from Table 2 are visualized in the dual-axis plot presented as Figure 2. This plot clearly delineates the performance of each clustering technique concerning both computational efficiency and path optimization.

**Figure 3:**
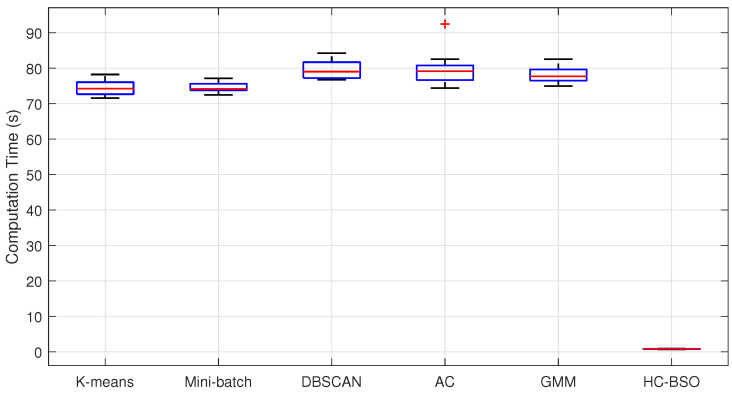
Box plots of computation times.

Figure 2 intuitively illustrates the trade-off between average computation time and average total path length for the different clustering techniques. However, mean values and CI alone do not provide a complete picture of algorithmic stability. To conduct a more in-depth analysis of the distribution characteristics and variability of computation times for each method, Figure 3 presents box plots of these times for the six clustering techniques.

As Figure 3 reveals, our proposed HC-BSO method demonstrates exceptional stability in terms of computation time. Its box plot, representing the interquartile range (IQR), is remarkably narrow and positioned near the origin. Furthermore, the median (indicated by the red line within the box) is substantially lower than those of all traditional methods. These observations suggest that our improved method consistently achieves very low computation times across the overwhelming majority of experimental runs, which is characterized by a highly concentrated data distribution and minimal variance. In contrast, the other clustering methods generally exhibit higher computation times, wider ranges of fluctuation, and a greater number of outliers, which could potentially compromise the real-time performance and reliability of multi-robot path planning. The DBSCAN and AC methods, in particular, display higher medians, broader data distributions, and pronounced outliers, suggesting inconsistent performance across different datasets. Although the GMM method demonstrates comparatively less fluctuation, some outliers are still present.

**Figure 4:**
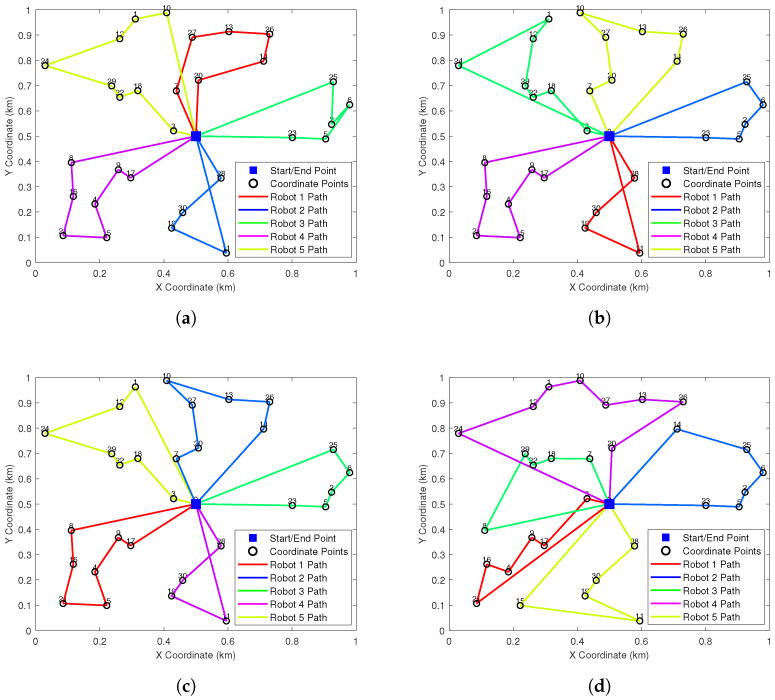

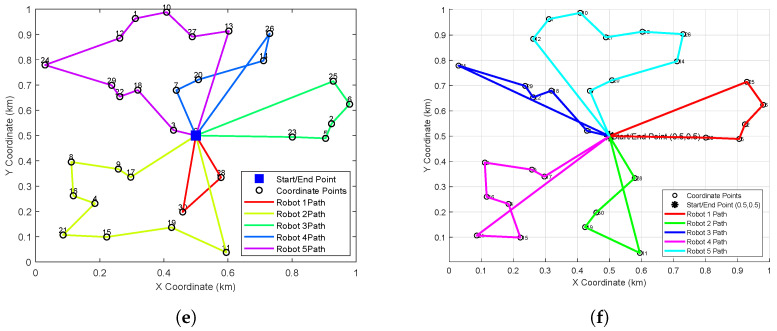
Comparison of the path planning route maps before and after the improvement. (**a**) K-Means. (**b**) Mini-Batch K-Means. (**c**) DBSCAN. (**d**) AC. (**e**) GMM. (**f**) HC-BSO.

Figure 4 illustrates the optimal path planning outcomes for five conventional clustering algorithms (subplots a–e) alongside those from our improved HC-BSO algorithm (subplot f). Inspection of subfigures a, d, and e reveals that the paths generated by these conventional methods often exhibit problematic intersections. Such path crossings are a concern as they can lead to conflicts among robots, potentially diminishing both task execution efficiency and overall operational safety. In stark contrast, the paths planned using our HC-BSO approach (subplot f) are entirely free of these crossings, thereby effectively precluding inter-robot conflicts.

**Figure 5:**
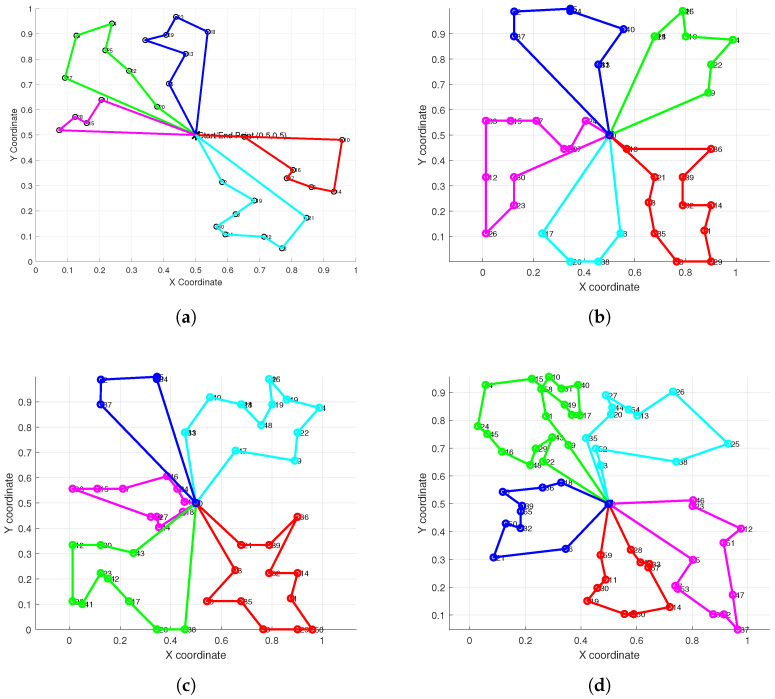

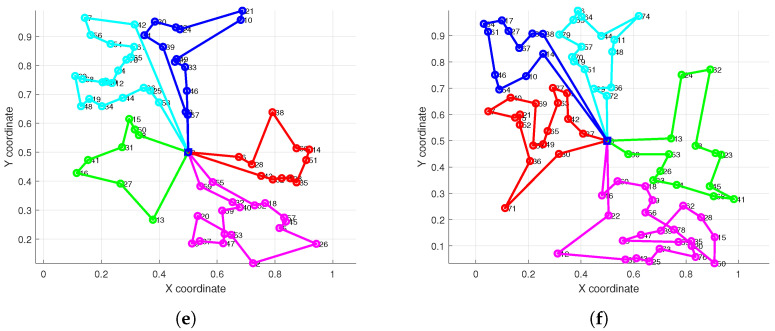
Experimental results across various test scenarios. (**a**) 30 task points. (**b**) 40 task points. (**c**) 50 task points. (**d**) 60 task points. (**e**) 70 task points. (**f**) 80 task points.

To comprehensively evaluate the performance and robustness of our improved method across tasks of varying scales and complexities, we designed several distinct test scenarios. Figure 5 showcases the path planning results generated by our improved method in six different test scenarios, where the number of task points was systematically increased from 30 to 80. These experiments were intended to verify the capability of the improved method to consistently generate effective, intersection-free path plans as the task scale increases.

The results presented in Figure 5 demonstrate that, across all six test scenarios, our proposed improved method successfully generated complete path plans for all robots that were free of mutual intersections, even as the number of task points increased. This indicates that our improved method is not only effective in isolated scenarios but also exhibits strong robustness and consistency when applied to tasks of varying complexities. Consequently, it reliably resolves the path intersection problem frequently encountered in multi-robot path planning.

### 5.3. Comparison with Other Swarm Intelligence Algorithms

To comprehensively evaluate the performance of our proposed HC-BSO method for multi-robot path planning, we benchmarked it against several swarm intelligence algorithms that are both representative and widely utilized in the optimization domain, particularly for path planning challenges. These include the FA, SA, GWO, ACO, and WOA. We selected these algorithms for comparison because they represent diverse optimization mechanisms and have demonstrated a certain capability in solving complex optimization problems of a similar nature. The experimental setup was configured as follows: the area of the experimental scenario was expanded to 100 km × 100 km. Each algorithm was independently executed 20 times to mitigate the influence of stochasticity, and the maximum number of iterations for each experimental run was set to 1000. Table 3 summarizes the performance comparison results between the HC-BSO algorithm and these competing algorithms.

The quantitative comparison results presented in Table 3 unequivocally demonstrate the comprehensive performance advantages of our proposed HC-BSO algorithm for multi-robot path planning tasks.

With respect to computational efficiency, the HC-BSO algorithm achieved an average computation time of 2.218 s. This significantly outperformed all benchmark algorithms, whose computation times ranged from 4.48 s to 31.76 s, underscoring its superior efficiency for large-scale or real-time applications.

In terms of path quality, as evaluated by the average total distance, the HC-BSO algorithm generated paths with an average total length of 674.4365 km, the minimum value observed across all considered algorithms. Among the comparative algorithms, GWO and SA yielded relatively favorable results (712.02 km and 718.26 km, respectively), yet their path lengths still exceeded those generated by HC-BSO. In stark contrast, ACO and FA produced paths with considerably greater total distances, specifically 1013.87 km and 1133.11 km, respectively.

A particularly salient advantage of the HC-BSO algorithm lies in its path conflict avoidance capabilities. Our HC-BSO algorithm achieved a consistent record of zero path conflicts across all experiments, whereas none of the benchmark algorithms entirely prevented path conflicts. Notably, FA, ACO, and WOA resulted in an average of over 10 conflicts. This finding underscores the reliability of the HC-BSO algorithm in facilitating safe and coordinated multi-robot operations.

**Figure 6:**
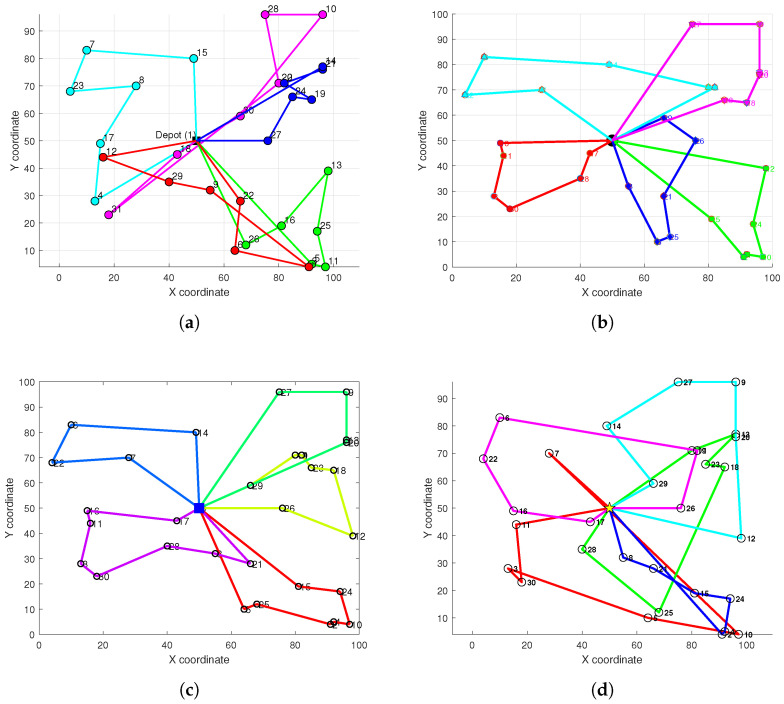

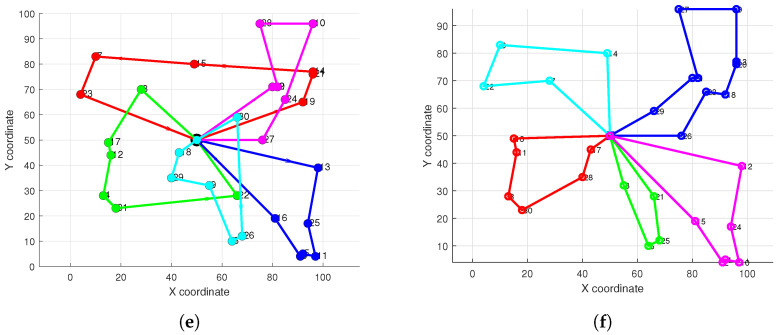
Performance comparison of the HC-BSO algorithm with other swarm intelligence algorithms. (**a**) FA. (**b**) SA. (**c**) GWO. (**d**) ACO. (**e**) WOA. (**f**) HC-BSO.

Figure 6 visually presents the path planning outcomes for the HC-BSO algorithm alongside those of the comparative methods within representative scenarios. These visualizations allow for a clearer observation of the path structures, overall configurations, and any instances of path intersections or conflicts produced by each algorithm, thereby complementing the quantitative analysis detailed in Table 3.

**Figure 7:**
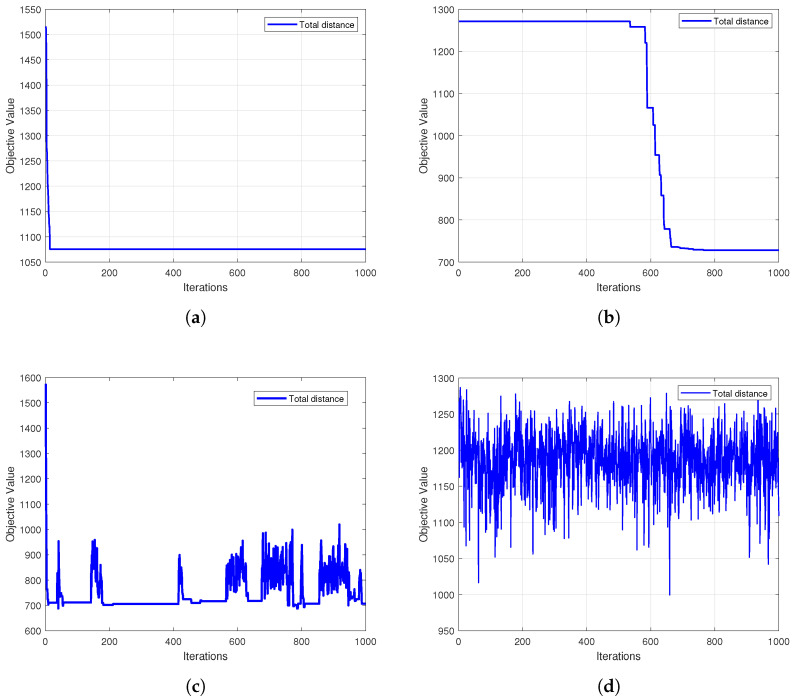

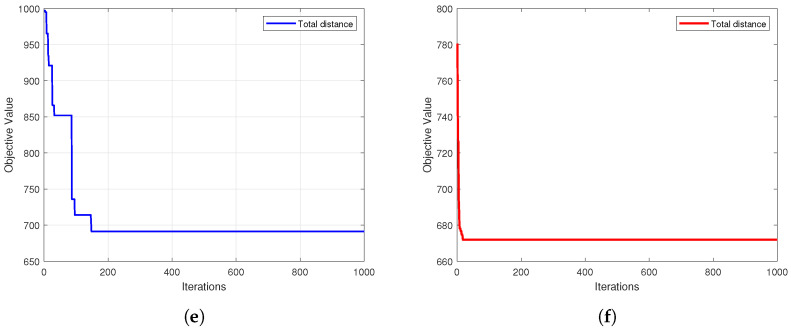
Convergence curve comparison between the HC-BSO algorithm and other swarm intelligence algorithms. (**a**) FA. (**b**) SA. (**c**) GWO. (**d**) ACO. (**e**) WOA. (**f**) HC-BSO.

The efficiency of an algorithm’s optimization process is another key indicator of its performance. Figure 7 further presents the convergence curves for the HC-BSO algorithm and the comparative algorithms, illustrating the evolution of their objective function values with an increasing number of iterations. Analyzing these convergence curves allows for an assessment of each algorithm’s search speed, convergence stability, and the quality of the final optimal solution attained.

Figure 7 displays the convergence curves for our HC-BSO algorithm (subplot f) alongside five comparative algorithms (subplots a–e) throughout their respective optimization processes. In each subplot, the vertical axis represents the objective function value, and the horizontal axis denotes the number of iterations. A closer inspection of these subplots reveals that while the FA algorithm (subplot a) initially demonstrates a rapid decrease, it converges to a relatively high objective function value. In contrast, our proposed HC-BSO algorithm not only exhibits faster convergence but also consistently achieves a superior objective function value compared to all benchmark methods. The benchmark algorithms (b), (c), (d), and (e) all demonstrate slower convergence and settle at less optimal final values than HC-BSO. Collectively, these six subplots affirm the efficacy of our modifications to the BSO algorithm, highlighting significant enhancements in both its optimization efficiency and the quality of the solutions obtained.

## 6. Discussion

The proposed HC-BSO algorithm, by incorporating a hybrid clustering strategy and a dynamic evolutionary mechanism, has demonstrated superior performance in MRPP tasks. To ensure the scientific rigor and fairness of the experimental conclusions, all algorithms were compared under a consistent hardware configuration, datasets of identical scale, and uniform parameter tuning protocols. The overall framework employs a synergistic approach, integrating two-stage clustering with dual evolution, thereby effectively balancing computational efficiency, path quality, and system robustness.

Specifically, during the task point partitioning phase, the proposed two-stage clustering strategy initially leverages Mini-Batch K-Means for rapid initial clustering, accelerating overall processing. Subsequently, DBSCAN refines these cluster structures and eliminates noise points through density-based analysis, enhancing the adaptability of the clustering outcomes to non-uniformly distributed data and anomalous task points. This hybrid clustering strategy effectively mitigates the inherent limitations of conventional K-Means, namely its sensitivity to initial centroid selection and its suboptimal performance in handling outliers. Experimental data indicate that, compared to traditional K-Means clustering, our method achieved an average computation time of merely 0.77 s—a reduction of approximately 98.98%. Furthermore, the total path distance was 6.42 km, representing an optimization exceeding 18%. These results markedly enhance the stability and rationality of the task assignment process.

In the search optimization stage, HC-BSO incorporates a dynamic population initialization mechanism alongside a dual evolutionary mechanism, which encompasses both global exploration and local perturbation. The dynamic population initialization guides the distribution of the initial solution space, thereby enhancing population diversity and the quality of initial solutions. This, in turn, accelerates the convergence of the subsequent evolutionary process. The dual evolutionary mechanism adeptly balances the breadth of global exploration with the depth of local perturbation. This not only augments the diversity of the solution space but also effectively prevents the algorithm from becoming ensnared in local optima, thereby expediting the refinement and convergence towards high-quality solutions. Experimental data demonstrate that this mechanism yields significant improvements in both total path distance optimization and computation time control.

Notably, to address multi-robot path conflicts, our approach employs a parallelized conflict detection and avoidance strategy. This allows for the rapid detection and elimination of potential conflicts concurrently with task assignment and path planning, ultimately achieving conflict-free path planning. This capability is paramount for practical multi-robot collaborative applications, as it markedly enhances the coherence of path planning and overall system safety.

Under fair experimental conditions, the substantial improvement in computational efficiency achieved by our method can be primarily attributed to several factors: First, the hybrid clustering method, by facilitating rapid initial partitioning and effective outlier removal, significantly reduces the computational load per clustering iteration and concurrently improves the quality of the initial task assignments. Second, dynamic population initialization enhances the quality of the initial solutions, thereby accelerating convergence during the evolutionary process. Finally, the dual evolutionary mechanism, while preserving solution diversity, curtails redundant search behaviors, which further diminishes the overall computational burden on the system. Both theoretical analysis and our experimental results indicate that, for large-scale MRPP tasks, the proposed method exhibits superior time complexity and operational efficiency.

The 95% CI derived from the experiments indicates that the proposed method exhibits significantly less variability in computation time (CI: [0.75,0.70] seconds) and path distance (CI: [6.39,6.45] km) when compared to the benchmark algorithms. This observation strongly substantiates the stability and robustness of our algorithm. Furthermore, these empirical findings lend strong support to the research innovations articulated in the introduction, underscoring a commendable alignment between the theoretical design of our method and its observed practical performance.

The outcomes of the performance comparison further substantiate the efficacy of the strategies discussed. In comparative evaluations against other prominent swarm intelligence algorithms, including FA, SA, ACO, GWO, and WOA, HC-BSO demonstrated superior performance across all key metrics. HC-BSO achieved an average computation time of 2.218 s, which is markedly lower than that of algorithms such as ACO (4.48 s) and FA (31.76 s), thereby exhibiting excellent computational efficiency. Regarding path quality, HC-BSO yielded the shortest average total distance (674.4365 km), representing a considerable improvement over GWO (712.02 km). More critically, the HC-BSO algorithm successfully navigated all test instances without generating any path conflicts. In contrast, the other algorithms commonly encountered path crossing issues to varying extents (e.g., SA: 4 instances, GWO: 2 instances, while both ACO and WOA exceeded 10 instances). Such conflicts could potentially lead to mission failure or compromise system safety in real-world deployments, thereby underscoring the distinct advantages of our proposed method in the context of multi-robot collaborative control.

Although the HC-BSO algorithm demonstrates commendable performance in MRPP tasks within static environments, it still encounters certain challenges when addressing dynamic settings or scenarios involving a very large number of tasks. For instance, the real-time generation of task points or dynamic changes in environmental obstacles might impose more stringent demands on the algorithm’s real-time responsiveness and adaptability. While the current method exhibits high computational efficiency, a substantial increase in the number of task points (e.g., exceeding several hundred) or frequent environmental fluctuations could still lead to a notable rise in computation time, thereby impacting its real-time performance. Furthermore, to maintain a focus on path optimization performance, the present study did not incorporate explicit obstacle avoidance strategies. This, to some extent, constrains the algorithm’s direct applicability in complex, real-world environments.

Future research endeavors could be directed towards several key areas: First, we could enhance the algorithm’s adaptive mechanisms for dynamic environments, for example, by introducing strategies for incremental task point updates and local replanning. Second, integrating efficient obstacle avoidance algorithms, such as Model Predictive Control (MPC) or Reinforcement Learning (RL), could improve path feasibility and safety. It is anticipated that these extensions will further broaden the applicability of the HC-BSO algorithm, allowing it to realize its full potential in practical scenarios, including industrial manufacturing, intelligent logistics, and emergency rescue operations.

## 7. Conclusions

This paper introduces HC-BSO, an optimized MRPP method rooted in an improved BSO algorithm. By integrating a hybrid clustering technique, which synergizes Mini-Batch K-Means with DBSCAN—and by incorporating dynamic population initialization alongside a dual evolutionary mechanism (encompassing exploration and perturbation), HC-BSO markedly enhances both the efficiency of task allocation and the quality of path planning. The hybrid clustering strategy, through its rapid initial partitioning and subsequent density-based refinement, bolsters the algorithm’s adaptability to complex task distributions. Concurrently, the dynamic evolutionary mechanism, balancing global exploration with local optimization, ensures the efficiency and robustness of the path planning outcomes.

Experimental results demonstrate that our proposed HC-BSO method outperforms conventional clustering approaches (such as K-Means, Mini-Batch K-Means, DBSCAN, AC, and GMM) and other established swarm intelligence algorithms (including FA, SA, GWO, ACO, and WOA) in terms of both average computation time and total path distance. Specifically, HC-BSO achieved an average computation time of 0.77 s, representing a reduction of approximately 98% compared to K-Means. The total path distance was minimized to 6.42 km, an improvement exceeding 18% relative to the K-Means method. Critically, HC-BSO accomplished these results with zero path conflicts, showcasing excellent coordination and safety.

Nevertheless, the application of HC-BSO in ultra-large-scale scenarios or highly dynamic environments warrants further optimization. Future research could profitably focus on the integration of dynamic obstacle avoidance mechanisms and the validation of the algorithm within real-world robotic systems, thereby aiming to further augment its practical utility.

## Figures and Tables

**Table 1:** Comparison of characteristics and applicability of five clustering techniques in MRPP.

Clustering	Type	Advantages	Disadvantages	Applicable Scenarios
K-Means	Center-based	Efficient, easy to implement	Sensitive to initial centers, affected by noise	Regular task distributions (e.g., warehouse scheduling)
Mini-Batch K-Means	Center-based	High efficiency, suitable for large datasets	Less stable, complex parameter tuning	Real-time large-scale tasks (e.g., dynamic logistics)
DBSCAN	Density-based	Robust, handles noise and complex shapes	High complexity, sensitive to parameters	Noisy, complex task distributions (e.g., urban rescue)
AC	Hierarchical	Hierarchical structure, multi-granularity	High computational demand, low efficiency	Multi-level task allocation (e.g., logistics network)
GMM	Probabilistic	Soft clustering, handles overlapping data	Requires pre-specifying components, prone to local optima	Overlapping, complex task distributions (e.g., dense urban environments)

**Table 2:** Consolidated performance statistics for clustering techniques.

Clustering Technique	Avg. Computation Time (s)	95% CI of Time (s)	Avg. Total Path Length (km)	95% CI of Distance (km)	Time Improvement (%)	Distance Improvement (%)
K-Means	75.79	[74.645, 76.941]	7.84	[7.456, 8.216]	−98.98%	−18.08%
Mini-Batch K-Means	76.93	[75.455, 78.403]	7.01	[6.849, 7.175]	−99.00%	−8.46%
DBSCAN	80.82	[79.636, 82.006]	7.16	[6.996, 7.336]	−99.05%	−10.43%
Hierarchical Clustering	78.71	[76.940, 80.478]	7.73	[7.457, 8.005]	−99.02%	−16.97%
GMM	78.35	[77.339, 79.361]	7.39	[7.105, 7.671]	−99.02%	−13.11%
HC-BSO	0.77	[0.744, 0.796]	6.42	[6.390, 6.448]	—	—

**Table 3:** Comparison of the improved BSO with other swarm intelligence algorithms.

Algorithm	Avg. Computation Time (s)	Avg. Total Distance (km)	Path Conflicts
FA	31.76	1133.11	>10
SA	12.54	718.26	4
GWO	21.46	712.02	2
ACO	4.48	1013.87	>10
WOA	4.69	771.83	>10
HC-BSO	2.218	674.4365	0

## Data Availability

Data will be made available on request.

## Abbreviations

The following abbreviations are used in this manuscript:

MRPP	Multi-Robot Path Planning
HC-BSO	Hybrid Clustering-Enhanced Brain Storm Optimization
BSO	Brain Storm Optimization
MRS	Multi-Robot System
DBSCAN	Density-Based Spatial Clustering of Applications with Noise
AC	Agglomerative Clustering
GMM	Gaussian Mixture Model
DWA	Dynamic Window Approach
GA	Genetic Algorithm
FA	Firefly Algorithm
SA	Simulated Annealing
PSO	Particle Swarm Optimization
GWO	Grey Wolf Optimizer
ACO	Ant Colony Optimization
WOA	Whale Optimization Algorithm
IWOA	Improved Whale Optimization Algorithm
RRT	Rapidly exploring Random Tree
CBS	Conflict-Based Search
MTSP	Multi-Traveling Salesman Problem
UAV	Unmanned Aerial Vehicle
CI	Confidence Intervals
IACO	Improved Ant Colony Optimization
IGWO	Improved Grey Wolf Optimization
ESCA	Equal-Sized K-Means Clustering Algorithm
AGA	Asynchronous Genetic Algorithm
AMA	Adaptive Memory Algorithm
SJSA	Swap-and-Judge Simulated Annealing
